# Underdispersion and overdispersion of traits in terrestrial snail communities on islands

**DOI:** 10.1002/ece3.1084

**Published:** 2014-04-26

**Authors:** Tina Astor, Joachim Strengbom, Matty P Berg, Lisette Lenoir, Bryndís Marteinsdóttir, Jan Bengtsson

**Affiliations:** 1 Department of Ecology, Swedish University of Agricultural SciencesP.O. Box 7044, Uppsala, SE-75007, Sweden; 2 Department of Ecology Science, Section Animal Ecology, VU University AmsterdamAmsterdam, 1081 HV, The Netherlands; 3 Department of Ecology, Environment and Plant Sciences, Stockholm UniversityStockholm, SE-106 91, Sweden

**Keywords:** Community assembly rules, convergence, divergence, environmental filtering, functional diversity, functional traits, limiting similarity

## Abstract

Understanding and disentangling different processes underlying the assembly and diversity of communities remains a key challenge in ecology. Species can assemble into communities either randomly or due to deterministic processes. Deterministic assembly leads to species being more similar (underdispersed) or more different (overdispersed) in certain traits than would be expected by chance. However, the relative importance of those processes is not well understood for many organisms, including terrestrial invertebrates. Based on knowledge of a broad range of species traits, we tested for the presence of trait underdispersion (indicating dispersal or environmental filtering) and trait overdispersion (indicating niche partitioning) and their relative importance in explaining land snail community composition on lake islands. The analysis of community assembly was performed using a functional diversity index (Rao's quadratic entropy) in combination with a null model approach. Regression analysis with the effect sizes of the assembly tests and environmental variables gave information on the strength of under- and overdispersion along environmental gradients. Additionally, we examined the link between community weighted mean trait values and environmental variables using a CWM-RDA. We found both trait underdispersion and trait overdispersion, but underdispersion (eight traits) was more frequently detected than overdispersion (two traits). Underdispersion was related to four environmental variables (tree cover, habitat diversity, productivity of ground vegetation, and location on an esker ridge). Our results show clear evidence for underdispersion in traits driven by environmental filtering, but no clear evidence for dispersal filtering. We did not find evidence for overdispersion of traits due to diet or body size, but overdispersion in shell shape may indicate niche differentiation between snail species driven by small-scale habitat heterogeneity. The use of species traits enabled us to identify key traits involved in snail community assembly and to detect the simultaneous occurrence of trait underdispersion and overdispersion.

## Introduction

How species assemble into communities has puzzled ecologists for decades. The basic processes shaping communities and their diversity are dispersal, drift, selection, and speciation, and their interactions (Vellend 2010). While species are added to a species pool via speciation and dispersal, their abundances are affected by random processes (drift), deterministic fitness differences (selection), and ongoing dispersal. Commonly, processes resulting in random patterns of community composition (Connor and Simberloff 1979), for example neutral performance of individuals (Hubbell 2001), are distinguished from processes resulting in deterministic or nonrandom patterns, which are often termed assembly rules (MacArthur and Levins 1967; Diamond 1975; Weiher and Keddy 1995). However, the relative importance of those processes in the assembly of communities and the underlying mechanisms are not well known for many ecosystems.

The assembly of communities is currently viewed as a hierarchical sequence of different filters acting on various spatial scales. The assumption of random assembly usually serves as null model to test for deterministic processes. Götzenberger et al. (2012) differentiated between *phylogenetic assembly* as a result of constraints due to long-term historic pattern of speciation, extinction and biogeographic migration, and *ecological assembly* comprising dispersal (both active and passive), abiotic and biotic processes. These processes are hypothesized to act at subsequently finer spatiotemporal scales (Zobel 1997), with local community composition representing the cumulative effect of all these processes. Communities that show a deterministic assembly pattern can comprise either species that are more similar (underdispersed/convergent) or more different (overdispersed/divergent) to each other than expected from a random distribution. While most previous studies on community assembly based on species occurrences or abundances can only detect one of those patterns, more recent studies based on functional traits have challenged this dichotomous view and show that both patterns can occur simultaneously (Cornwell and Ackerly 2009; Ingram and Shurin 2009; Naaf and Wulf 2012).

If species are primarily sorted by a common environmental filter, they should have certain traits in common that enable them to sustain the prevailing environmental conditions, that is be underdispersed in those traits (Weiher and Keddy 1995; Fukami et al. 2005; Silva and Batalha 2008). Dispersal limitation (which often is considered as part of the environmental filter) is another mechanism resulting in underdispersion. Trait underdispersion can also occur due to predation (Zaret 1980; Abrams and Chen 2002; Chase et al. 2002), natural enemies such as pathogenes (Mitchell and Power 2003), and competitive exclusion in the presence of a common limiting factor (Mayfield and Levine 2010).

It has frequently been shown that traits are involved in maintaining species diversity through niche partitioning (Stubbs and Wilson 2004; Kraft et al. 2008; Mason et al. 2012). If species are sufficiently different (i.e., exhibit overdispersion) in traits related to resource requirements and resource acquisition they are more likely to coexist. This was the basis of the classical theory of limiting similarity (MacArthur and Levins 1967; Diamond 1975). It is likely that various filters will operate and impact community assembly simultaneously, but it is currently unclear under which conditions each filter predominates and what the results of each filter may actually be.

The aim of our study was to examine the relative importance of underdispersion and overdispersion in 12 functional traits of terrestrial snails on lake islands. For convenience, we use the term trait for all our species characters (including diet and niche occurrences) although the term is commonly restricted to morphological, phenological, or phenotypic features that impact the fitness of organisms (Violle et al. 2007). We hypothesize that traits related to resource acquisition and utilization or microhabitat occurrence will exhibit overdispersion, whereas traits that are related to dispersal ability and tolerance to abiotic conditions will exhibit underdispersion (see Table 1 for detailed predictions). Another aim was to reveal the environmental gradients that are related to shifts in traits across islands and underlie the observed assembly pattern. Island size and factors changing with island size were expected to play a crucial role because island area has been shown to be positively related to species richness in this system (Nilsson et al. 1988). We only consider ecological assembly processes in our study because the islands in our study system are relatively young, not older than a few thousand years, and are of similar ages. To test our hypotheses, we reanalyzed data from a former study on species area and habitat heterogeneity relationships (Nilsson et al. 1988) in a system of currently undisturbed forested islands situated in Lake Mälaren, Sweden using a trait-based approach. Traits were not measured on-site in the original study, which is why we rely on published information on terrestrial snails from an extensive snail database (Falkner et al. 2001).

**Table 1:** List of the selected traits from the database on Shelled Gastropoda of Western Europe (Falkner et al. 2001) and filters that are expected to act on them. Observe that some traits can be affected by several filters. See also text for explanation and justification of predictions

Filter	Traits	Pattern
Dispersal (+establishment)	Shell size (mss)	Underdispersion
	Number of offspring (noo)	
	Age at maturity (mat)	
	Reproduction mode (rep)	
	Number of reproduction periods (norp)	
Environment	Survival of dry period (sdp)	Underdispersion
	Humidity preference (hpr)	
	Inundation tolerance (int)	
	Ecosystem occurrence (eco)	
	Microhabitat occurrence (micro)	
Niche partitioning	Shell size (mss)	Overdispersion
	Shell shape (ssh)	
	Diet	
	Microhabitat occurrence (micro)	
	Humidity preference (hpr)	

## Material and Methods

### Study area

**Figure 1:**
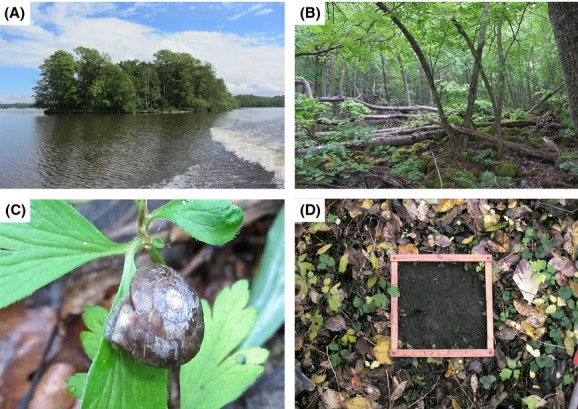
(A) The smallest island, Benklädet (0.7 ha), covered with mixed deciduous forest. (B) Scree in mixed deciduous forest on the island Alholmen (9.4 ha). (C) The snail *Helicigona lapicida* on Alholmen. (D) Snail sampling square (0.1 m^2^) showing how the litter and uppermost soil layers were collected. The material was placed in plastic bags, brought to the laboratory, dried and sieved, after which snails were extracted by hand sorting.

Data on terrestrial snail species composition and abundance were obtained for 17 islands in the central part of lake Mälaren, Sweden in 1981 (Nilsson et al. 1988). The islands are located within an area of approximately 1062 km^2^, they have been formed by land uplift and are, depending on island height, 1000–4000 years old. They are covered with mature unmanaged forest, and their edaphic conditions are heterogeneous with the proportion of morainic soil, exposure of bedrock and sediments varying among the islands (Fig. 1). Some islands (mostly smaller ones) are part of an esker ridge (Högholmen, Hargen, Grävlingen, Benklädet, and Räfsgarn) with a more lime-rich, coarse-grained soil that is highly permeable (Kers 1978). The islands differ in size, distance to the mainland, habitat diversity, plant diversity, amount of deciduous and coniferous forest, and tree cover, creating several environmental gradients that influence land snails (Nilsson et al. 1988).

On each island, ground-living snails were sampled on five occasions from May to September 1981. Both living and recently dead snails (empty fresh shells) were collected because empty shells represent individuals from the year of the sampling or the year before (due to rapid decomposition older shells are not present) and can therefore be considered to represent the current community. The snails were sampled by collecting litter and the uppermost soil layer from five to seven randomly placed 0.1 m^2^ squares within 10 × 10 m plots. The counts from each small square were lumped together to give one count per species for each plot. The number of plots (10 × 10 m) on the islands varied from one on the smallest islands to four on the largest ones (see Table A9 in the supplementary material and Nilsson et al. 1988 for more details on the sampling). The litter samples were dried at 50°C, and the snails were hand-sorted after sieving (Nilsson et al. 1988). Slugs (nonshelled Gastropods) were not included in the sampling campaign, because they could not be sampled adequately with the same methods that were used for the sampling of shelled snails. In total, 33 snail species were found (Appendix S1, Table A1). The number of species found per island ranged from 9 to 26. A jackknife estimate of the number of species revealed that on average, two species per island were not included in the samples (see Nilsson et al. 1988). As our trait analyses are based on abundance-weighted trait values, missing a few rare species should not influence our results.

### Selection and use of traits

Trait information was taken from a database of shelled snails containing information on traits ranging from macro- and microhabitat occurrences to physiological and biological traits of 270 European snail species (Falkner et al. 2001). To our knowledge, this is currently the most comprehensive collection of trait data available for snails. The database also comprises information on the potential range of the trait values within species. Even though traits such as shell size or shape may vary under different environmental conditions, the difference in trait values for the traits we selected is larger between species than within species, which justifies the use of such published traits in our analysis.

From the species present in the former study, we excluded *Succinea* sp. because it was not determined to species level. For the remaining species, we selected traits that are related to dispersal, environmental tolerance, and niche differentiation (Table 1).

Dispersal ability and abiotic environmental conditions both can lead to a reduction in trait range (i.e., trait underdispersion). Together they determine whether a species can colonize an island, because to be present a species should have to be both able to reach the island, and have the right set of traits to be able to survive the abiotic conditions. Important traits here are dispersal traits, tolerance traits and habitat occurrences (reflecting the environmental conditions needed for survival). During the establishment phase, traits related to reproduction can also be important.

Large-bodied snail species are often found to be more mobile and better dispersers (Sutherland et al. 2000; Brouwers and Newton 2009). However, snails are poor active dispersers (Schilthuizen and Lombaerts 1994) and even larger species, such as *Arianta arbustorum*, *Cepaea nemoralis,* or *Cepaea hortensis* do not disperse more than 12–86 m per year (Day and Dowdeswell 1968; Baur and Baur 1993). Instead, passive dispersal or accidental dispersal by birds has been suggested as the main dispersal mechanism for terrestrial snails (Schilthuizen and Lombaerts 1994; Gittenberger et al. 2006). In case of passive dispersal, small-bodied species may be more easily dispersed (Hausdorf 2000). Indeed, small shell size has been recognized as a dispersal trait for terrestrial snails (Vagvolgyi 1975). Apart from shell size, there is hardly any information available on which traits are related to the dispersal ability of snails (but see Baur 1991 for intraspecific influence of life history traits on range expansion). Studies from various animal groups suggest that species with high reproductive potential, for example, number of offspring (Stevens et al. 2012), broad tolerance to abiotic conditions (Martin and Sommer 2004), and generalist species (Baur and Bengtsson 1987; Jocque et al. 2010) are more likely to successfully establish a population on an empty site; hypotheses related to the classical idea of *r*-selected species (MacArthur and Wilson 1967). Humidity is an important abiotic factor influencing abundance and diversity of snails (Martin and Sommer 2004). Hence, we included the traits humidity preference and tolerance to dry conditions. Shell size and shell shape could be constrained by environmental factors (Schamp et al. 2010) and habitat structure (Cain 1977; Heller 1987) and are regarded as traits indicating environmental filtering (for detailed predictions see Table 1).

Diet (Bowers and Brown 1982), shell size (Chiba 1996; Lee and Silliman 2006), and shell shape (Cain and Cowie 1978; Cameron and Cook 1989) have been found to be involved in competition and niche differentiation. Therefore, if competition plays a major role, it is likely that communities exhibit overdispersion in those traits. Body size has been linked to niche partitioning via specialization on different resources (Bowers and Brown 1982). Shell shape is indicative of the preferred microhabitats (Cain and Cowie 1978; Cameron and Cook 1989), as snails with flat shells tend to prefer horizontal structured habitats such as litter, whereas elongated snails tend to prefer vertical surfaces (Cain and Cowie 1978) such as tree trunks. In addition, microhabitat occurrences reflect where the species prefer to live on a small scale, such as on trees, in the litter layer or on mosses. At this, small-scale species can potentially interact and compete which might lead to niche partitioning (for detailed predictions see Table 1).

We used the information in Falkner et al. (2001) to calculate average values for each species and trait. Each trait in the database consists of several categories wherein each entry describes the degree of association between a species and the trait category. The degree of association can take values from 0 to 3, with 0 defined as no association, one as weak association, two as moderate association and three as strong association to the respective category. This means that the categories are not always mutually exclusive, but have a fuzzy coding structure (see Appendix S1, Table A8.2 for an example). The number of reproduction periods was calculated by counting the occurrences in the corresponding main reproduction period categories within a year (Appendix S1, Table A8.1**)**. As we did not use all the food-type categories present in the database due to redundancy among some categories, we could not keep the original scoring but converted the categories to a binary multichoice variable (Appendix S1, Table A4). The same was carried out for the ecosystem occurrence and microhabitat occurrence (Appendix S1, Table A5 and Table A6). For all other traits, we calculated a mean trait value from the fuzzy coded entries (see Appendix S1, Table A8b for an example). In the original data set, carnivorous and saprophagous species were grouped into one category. We separated this category into two new categories because carnivory and saprophagy are two different strategies. To the category “carnivorous,” we assigned species for which carnivorous behavior is reported in the literature (Taylor 1914; Rondelaud 1977; Badie and Rondelaud 1985). Of these, only *Zonitoides nitidus* is an efficient active predator (Rondelaud 1978). All other species in this category can be considered as facultative carnivores (Barker and Efford 2004). Also note that food niche breadth might be underestimated for some species because many macrodetritivores including snails do not eat primarily pure litter, but ingest the microbial biofilm attached to it as an important part of their diet (Hax and Golladay 1993).

### Environmental variables

Twelve environmental variables (Table 2) were used to test for a link between traits and environmental variables. The theory of island biogeography (MacArthur and Wilson 1967) considers island area and distance to the mainland to be two central factors affecting the number of species on an island. Those might also affect the functional richness and composition. Distance to the mainland is an isolation measure and affects the immigration rate, whereas area affects the probability of persistence, that is, extinction rate on an island. We also considered the distance to the next largest island as an additional measure for isolation. Land snail species richness has previously been found to be related to plant diversity (Barker and Mayhill 1999). As humidity has also been shown to be important for species richness and abundance of snails (Martin and Sommer 2004), we included a habitat wetness index based on indicator plants of the ground vegetation (Nilsson et al. 1988). In addition, we tested several environmental variables that might reflect habitat quality and heterogeneity (leaf dry matter content, basal area of deciduous trees, number of habitats per island, woody plant richness, location on esker ridge, and a measure for productivity based on indicator plants of the ground vegetation (Nilsson et al. 1988). Indices like the wetness and productivity index are based on indicator species as proxies for environmental variables. Therefore, they have limitations because species not always are found at their environmental optimum. However, these proxies may still give a good indication of major differences in humidity and productivity between islands, in the absence of more detailed information. Leaf dry matter content might be important for snails that feed on leaves or leaf litter. Leaves with a high LDMC are less palatable compared to leaves with a low LDMC. Average leaf dry matter content (LDMC; mg/g) of tree species was compiled from data gathered at 17 other forest sites around Lake Mälaren Sweden in 2008. At each site, 12 leaves from all species of trees and shrubs were collected in spring and autumn and LDMC measured in the laboratory following the guidelines from Cornelissen et al. (2003). Using data for each tree and shrub species, an average LDMC was calculated for each sampling plot on each island. The remaining variables were taken from Nilsson et al. (1988).

**Table 2:** List of environmental predictor variables included in the CWM-RDA and regression analysis. For more detailed description of the variables, see Nilsson et al. (1988)

Environmental predictor variables	Range	Source
Island area [ha]	0.6–74.3	(Nilsson et al. 1988)
Distance to the mainland [m]	200–4050	(Nilsson et al. 1988)
Distance to the next largest island [m]	50–1650	(Nilsson et al. 1988)
Average tree cover [%]^1^	64.4–97.5	(Nilsson et al. 1988)
Woody plant richness	19–23	(Nilsson et al. 1988)
Number of habitats per island^2^	2–7	(Nilsson et al. 1988)
Mean basal area of deciduous trees (BADT) [% of living basal area]	53.65–98.87	(Nilsson et al. 1988)
Productivity of ground vegetation^3^	0–14.70	(Nilsson et al. 1988)
Wetness index of ground vegetation^3^	0–29.70	(Nilsson et al. 1988)
Leaf dry matter content (LDMC) [mg/g]	259.7–312.1	
Esker ridge	0 or 1	

1 Tree cover was estimated for each island as the mean vertical projection of the canopy (see Nilsson et al. 1988).

2 From the 19 habitat types that were previously determined by Nilsson et al. (1988).

3 Mean of the number of indicator species found in the plant sampling square divided by the total number of species in the square (Nilsson et al. 1988).

### Statistics

#### Trait underdispersion and overdispersion

Functional diversity comprises of three components: functional richness, functional evenness, and functional divergence (Mason et al. 2005; Villeger et al. 2008). There is an ongoing debate on which component of functional diversity does best describe community assembly. In contrast to functional evenness, functional richness and divergence have often been shown to be powerful components for detecting community assembly (Mouchet et al. 2010; Mason et al. 2012). Rao's quadratic entropy (Rao, henceforth) combines functional richness and divergence. When calculated with species occurrences, it resembles functional richness. When calculated with abundances, and compared to expected values under null models that randomize species abundances within communities, it becomes a pure functional divergence index (Mason et al. 2008). It is currently the only index which can partition regional functional diversity (*γ*-Rao) into within community (*α*-Rao) and among community (*β*-Rao) components, it can be calculated for single traits as well as for multiple traits, and it can take into account species abundances. Mouchet et al. (2010) showed that compared to other indices, it is only weakly related to species richness, but able to detect assembly pattern.

For each trait and sampling plot, we calculated Rao (both with species occurrences and abundances) and then averaged the values per island, resulting in 17 island-wise Rao measures (*α*-Rao) for each trait. In addition, we calculated the abundance weighted *β*- and *γ*-Rao for each trait using the additive approach by de Bello et al. 2009 (Appendix S1, Eq. A1, A3). Rao can also be calculated for multiple traits by summing the dissimilarities for each trait (Appendix S1, Eq. A2). This was carried out for the categories of diet and niche occurrences.

When examining various species diversity indices, Jost (2007) found that *β*-diversity values are always lower than expected because they are strongly dependent on *α*. If *α* becomes very large, *β* automatically approaches zero even if the communities are considerably dissimilar in species composition. Jost (2007) proposed a correction method based on equivalent numbers of *α*, *β,* and *γ* that makes comparison of *α*- and *β*-diversity possible. This approach was extended for the calculation of functional diversity (de Bello et al. 2010) (Appendix S1, Eq. A4–6), and we applied this correction method for the comparison of *α*- and *β*-Rao with *α*- and *β*-species richness and Simpson diversity.

#### Null models

We used a combination of different null models and Rao measures to compare the observed *α*-Rao with the expected *α*-Rao under random community assembly. A significantly higher than expected Rao indicates trait overdispersion, whereas a significantly lower Rao indicates trait underdispersion. To test for deviations from random assembly, we used three null models and calculated the standard effect size (SES; Gotelli and McCabe 2002) as (observed *α*-Rao minus mean of expected *α*-Rao) divided by standard deviation of expected *α*-Rao. The observed and expected values were compared, and the significance was tested with one-sided permutation tests (with 999 randomizations) using the function “as.randtest” of the package “ade4” (Dray and Dufour 2007). In one-tailed null model tests, observed values of SES < 1.55 (underdispersion) or >1.55 (overdispersion) indicate significant (*α* = 0.05) assembly pattern. In the first null model, we randomized communities (species x plots matrix) by reshuffling the species identity among islands while keeping the same number of species per site and the same total species occurrence frequency in the whole region and calculated the abundance-weighted *α*-Rao for each random community. This represents the original Rao index comprising both functional richness and functional divergence. The randomization procedure was carried out with the trial swap method of Miklos and Podani (2004) implemented in R (R core team) with the “randomizeMatrix” function of the package “picante” (Kembel et al. 2010) with 999 randomizations. For the second null model, we randomized the abundances among species *within* communities and calculated the abundance-weighted Rao. This converts the Rao into a pure divergence component. For the third null model, we again used the trial swap randomization, but calculated the Rao based on species occurrences (presence/absence) only. This resembles the functional richness component.

#### Environmental gradients

To examine relationships between traits that were significant in the assembly test and the environmental variables, we conducted a community weighted mean redundancy analysis (hereafter referred to as CWM-RDA). This procedure is useful to reveal changes in average trait expressions of communities along environmental gradients (Kleyer et al. 2012). First, a plot by trait matrix was created by averaging the trait values of all species per plot weighted by their abundances. Those values are CWM trait values (Garnier et al. 2007). We then used the CWMs constrained by the environmental variables in the RDA. The variable “ESKER” was coded as factor with two levels (1: located on the ridge and 0: not located on the ridge). To clarify toward which end of the environmental gradients over/underdispersion gets stronger, we performed linear regressions (Kruskal–Wallis rank sum test for variable ESKER) with the standard effect sizes of the traits that turned out to be significant in the assembly analysis (from second model, above) as dependent variables, and each environmental variable as explanatory variables. By combining both results, we obtained information on which part of the trait values is affected, and toward which end of the environmental gradients over/underdispersion gets stronger.

## Results

### Diversity Partitioning

**Figure 2:**
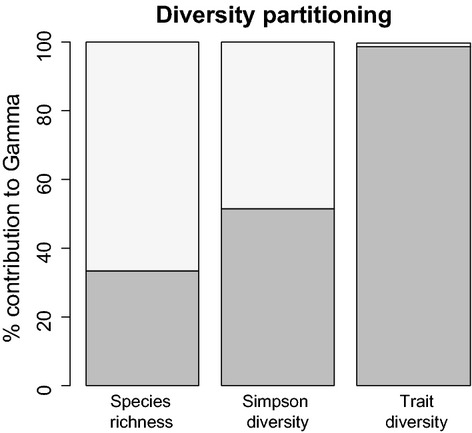
Percentage of *γ*-diversity (*y*-axis) accounted for by local *α*-diversity (dark gray) and between island *β*-diversity (light gray) shown for species (species richness), Simpson species diversity, and Rao trait diversity averaged over all traits: *α*-species richness = 66.6%; *β*-species richness = 33.4%; *α-*Simpson diversity = 51.5%; *β*-Simpson diversity = 48.5%; *α*-Rao = 98.8%; *β*-Rao = 1.2%.

All the traits analyzed showed a considerably higher *α*-Rao than *β*-Rao with averages of 99% and 1%, respectively. Using the Simpson diversity index, *β*-species diversity makes up almost half of the regional diversity (49%). The turnover of species between islands made up two-thirds (67%) of the regional species richness (Fig. 2). Hence, while species diversity varied among islands, trait diversity varied substantially less, and most islands contained most of the variation in trait composition.

### Trait underdispersion and overdispersion

With the first procedure, using the trial swap algorithm in combination with the abundance-weighted Rao, we identified six traits to be significantly underdispersed (humidity preference, ecosystem occurrence, max. shell size, survival of dry period, number of offspring, and age at maturity). As we hypothesized, these traits are related to dispersal and environmental filtering. No trait was found to be significantly overdispersed (Table 3). The pure divergence component, examined with the second procedure, identified two additional traits (reproduction mode and microhabitat occurrence) as significantly underdispersed and two traits (number of reproduction periods and shell shape) as significantly overdispered (Table 3). Shell shape was a trait we hypothesized to be overdispersed because it reflects preferred microhabitats of different structure and might therefore be involved in niche partitioning. With the third procedure, testing for the functional richness component, no significant under- or overdispersion could be detected (Table 3). In summary, the functional divergence component examined with the second procedure was most successful in refuting the hypothesis of random assembly patterns.

**Table 3:** Standard effect sizes for each trait from three different assembly tests. Significance was tested with one-tailed Monte Carlo tests SES < 1.55 indicates significant underdispersion and SES > 1.55 indicates significant overdispersion (*P* values are given in parenthesis). SES_div_: divergence component; abundances randomized *within* communities; SES_ric_: richness component; trial swap randomization and Rao calculated with species occurrences; SES: trial swap randomization and Rao calculated with abundances; Traits are ordered according to SES_div_ from significant overdispersion (top) to significant underdispersion (bottom). Bold figures indicate significance

	SES_div_	SES_ric_	SES
Number of reproduction periods	**2.15 (0.027)**	−0.80 (0.224)	1.43 (0.084)
Shell shape	**1.64 (0.058)**	0.004 (0.509)	−0.04 (0.476)
Food preference	−1.00 (0.157)	−1.19 (0.115)	0.59 (0.263)
Inundation tolerance	−1.54 (0.065)	−0.71 (0.229)	0.20 (0.431)
Reproduction mode	−**2.80 (0.001)**	−0.09 (0.484)	−0.86 (0.202)
Humidity preference	−**3.14 (0.001)**	−1.09 (0.133)	−**2.28 (0.006)**
Ecosystem occurence	−**4.67 (0.001)**	−1.40 (0.076)	−**2.22 (0.011)**
Max shell size	−**5.21 (0.001)**	−0.07 (0.492)	−**1.77 (0.027)**
Survival of dry period	−**5.65 (0.001)**	−0.65 (0.256)	−**2.83 (0.001)**
Number of offspring	−**5.83 (0.001)**	−0.47 (0.319)	−**3.45 (0.001)**
Microhabitat occurence	−**6.25 (0.001)**	−1.05 (0.143)	−0.90 (0.199)
Age at maturity	−**6.40 (0.001)**	−0.14 (0.42)	−**2.25 (0.005)**

### Environmental gradients

**Figure 3:**
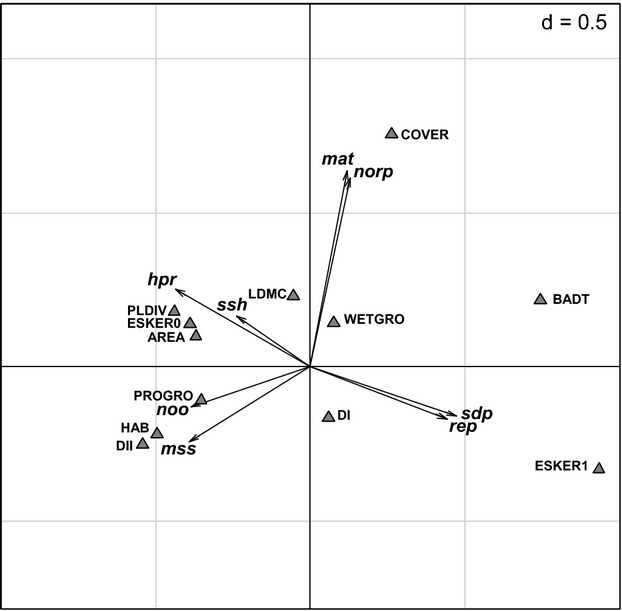
CWM-RDA with significant traits from the assembly analysis. Environmental variables (gray triangles) explained 76.3% of the total variance in community traits, and the first two axes explained 50.4 and 34.8% of this explained variation, respectively. PROGRO, productivity of ground vegetation; BADT, basal area of deciduous trees; DI, distance to the mainland; DII, distance to the next largest island; HAB, number of habitats, AREA, island area; LDMC, leaf dry matter content; WETGRO, wetness index of ground vegetation; PLDIV, woody plant diversity; COVER, tree cover; ESKER, esker ridge; sdp, survival of dry period; hpr, humidity preference; mat, age at maturity; noo, number of offspring; mss, max shell size; ssh, shell shape; norp, number of reproduction periods; rep, reproduction mode.

The environmental variables explained 76.3% of the total variance in community traits in the CWM-RDA, and the first two axes explained 50.4 and 34.8% of this variance, respectively. Location on the esker had the highest scores on the first axis, followed by basal area of deciduous trees, distance to the nearest large island, number of habitats, plant diversity, productivity of ground vegetation and island area (Fig. 3, Table 4). On the second axis, tree cover was the most important variable (Fig. 3, Table 4). Number of offspring and shell size were positively related to the distance to the next largest island and habitat diversity and negatively to tree cover and basal area of deciduous trees. Humidity preference and shell shape were positively related to woody plant diversity and area. Age at maturity and number of reproduction periods were mainly related to tree cover. Survival of dry period and reproduction mode were mainly related to location on the esker (Fig. 3, Table 4).

**Table 4:** Canonical correlations between each environmental variable (centroids for the factor variable ESKER) and the two main axes of the CWM-RDA. The environmental variables together explain 76.5% of the variance. Axes 1 and 2 explain 50.4 and 34.8% of this explained variance

	Axis 1	Axis 2
Tot. tree cover (COVER)	0.26	0.75
Woody plant diversity (PLDIV)	−0.44	0.18
Island area (AREA)	−0.37	0.10
Distance to the mainland (DI)	0.06	−0.17
Distance to the closest large island (DII)	−0.54	−0.25
Basal area of deciduous trees (BADT)	0.74	0.21
Number of habitats (HAB)	−0.50	−0.22
Leaf dry matter content (LDMC)	−0.06	0.23
Wetness index of ground vegetation (WETGRO)	0.08	0.14
Productivity of ground vegetation (PROGRO)	−0.35	−0.11
Nonesker (ESKER 0)	−0.39	0.14
Esker (ESKER 1)	0.93	−0.33

**Figure 4:**
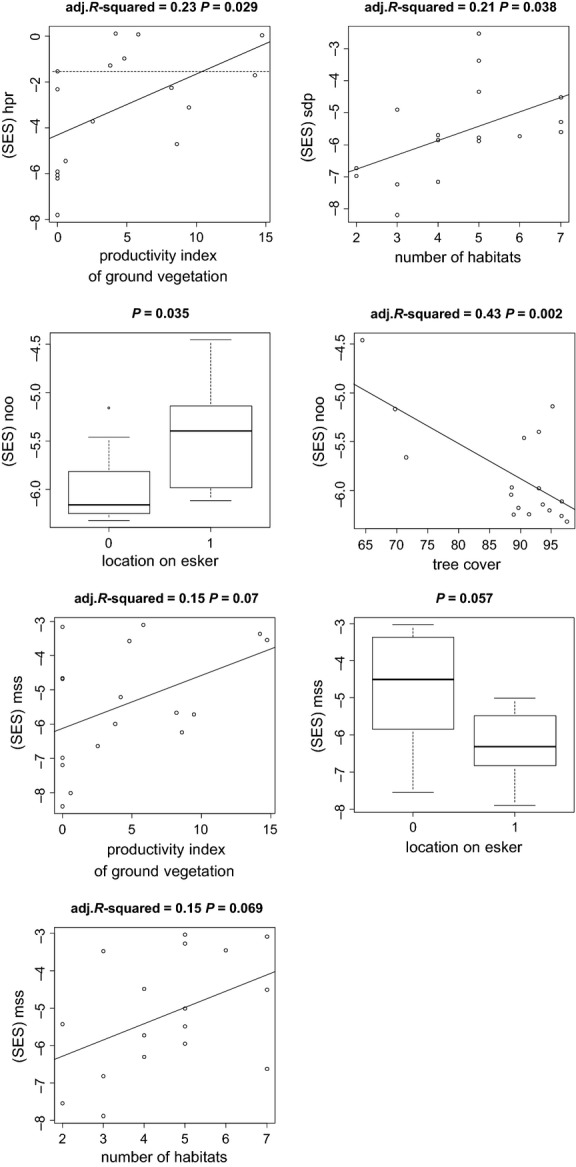
Results of regression analyses (Kruskal Wallis rank sum test for the factor variable ESKER) visualizing changes in the standard effect sizes across the environmental gradients for four traits. In those traits, the increase in underdispersion coincides with a shift in mean trait values (see CWM-RDA, Fig. 3). On the *y*-axis, values below zero represent underdispersion (with values <−1.55 being significant, which is marked by a dashed line).

Regression analysis with the effect sizes of those traits that were significant in the assembly analysis and the environmental variables revealed that the strength of underdispersion is affected by tree cover (for humidity preference, survival of dry period, number of offspring, reproduction mode, microhabitat occurrence, and ecosystem occurrence), productivity of ground vegetation (for shell size, and humidity preference), habitat diversity (for shell size), and location on esker ridge (for shell size and number of offspring). The strength of overdispersion in shell shape was affected by tree cover, productivity of ground vegetation, and distance to the next largest island (see Fig. A1 in the supplementary material for more details). For four traits, the strength of underdispersion (Fig. 4) coincided with a shift in mean trait values (Fig. 3). Species on islands with lower productivity were more strongly underdispersed and converged toward low average humidity preference and small average shell size. Species on islands with lower habitat diversity were more strongly underdispersed and converged toward low average survival of dry period and small shell size. Species on nonesker islands were more strongly underdispersed in number of offspring and converged toward a low average number of offspring, whereas species on esker islands were more strongly underdispersed in shell size and converged toward small shell size. Finally, species on islands with high tree cover were more strongly underdispersed and converged toward low number of offspring.

## Discussion

We provide evidence for the simultaneous occurrence of trait underdispersion and trait overdispersion as driving forces for the assembly processes of communities of terrestrial soil invertebrates. This has recently also been found in studies of plant communities (Cornwell and Ackerly 2009; Naaf and Wulf 2012) and aquatic ecosystems (Ingram and Shurin 2009). Trait underdispersion was relatively more important than overdispersion, being found in eight versus two traits, respectively.

In accordance with our hypothesis, we found that traits related to dispersal or to tolerance to environmental factors are underdispersed. Maximum shell size is clearly important for dispersal but can also be filtered by the environment when there are long climatic gradients (which is not the case in our study, however). Ecosystem- and microhabitat occurrence is an indicator of habitat requirements and is therefore also part of the environmental filter. Age at maturity and number of offspring can be important for a successful colonization of uninhabited islands, and thus linked to dispersal and environmental filtering. However, to conclude that the underdispersion is caused by dispersal constraints, the traits involved also need to be related to some measure of isolation. In the graphical representation of the CWM-RDA, maximum shell size and number of offspring are positively associated to the environmental variable *distance to the next largest island*, indicating changes related to isolation. Mean shell size and number of offspring also increase toward increasing productivity of ground vegetation and habitat diversity, indicating that larger species will be more likely to be found in areas with high habitat diversity. Survival of dry periods and humidity preference represent the snails’ tolerance/preference to abiotic conditions (drought). The underdispersion in those two traits clearly indicates the occurrence of environmental filtering. As shown in Fig. 3, a high survival of dry periods was related to small island size. The small islands also tend to have drier soils because most of them are located on the esker ridge. Given the age of the islands in the order of 1000–4000 years, it is likely that the snails had enough time for colonization. This suggests that the snail communities might not be dispersal limited and that abiotic environmental factors might be the main reason for trait underdispersion in this system. When niche partitioning is a driving mechanism in determining community assembly, traits that are related to resource requirements and acquisition are predicted to exhibit overdispersion to minimize similarity between species (MacArthur and Levins 1967; Diamond 1975; Wilson and Stubbs 2012). In accordance with our hypothesis, shell shape showed overdispersion. This could be an indication for niche partitioning because species with different shell shape prefer differently structured microhabitats. However, we found no evidence for niche separation according to shell size or diet in the present communities. In agreement with these results, resource limitation, that is, food limitation (Hatziioannou et al. 1994) and competition (Solem 1985; Cook 2008), are often considered to play a minor role in terrestrial snail communities.

The dominance of underdispersion or overdispersion could be influenced both by the spatial scale and the range of the environmental gradient. In our study, trait underdispersion was more common than trait overdispersion, which is in accordance with Freschet et al. (2011) who found a general prevalence of underdispersion in plant communities across spatial scales (local to global) and ecosystems (including most major biomes of the earth), but in contrast to the results of a meta-analysis on assembly pattern of plant communities by Götzenberger et al. (2012) who reported that trait overdispersion was more common than underdispersion. Moreover, Götzenberger et al. (2012) found that overdispersion tended to occur more often in studies covering small spatial scales. Although it may be difficult to compare studies conducted at different spatial scales, our findings contradict this result, as we found dominance of underdispersion despite that our study is conducted over a limited spatial scale (ca. 1000 km^2^) and over a relatively short environmental gradient (indicated by low turnover in traits, 1.2%, between islands compared to species composition). One possible reason might be that competition seems to be less important for snails (Solem 1985; Cook 2008).

Our study contributes to the growing body of literature on trait-based community assembly of organisms other than plants and it is unique in terms of the broad range of traits we used to make a priori predictions of the outcome of assembly tests. Further, we analyzed each trait individually rather than grouping many traits into one measure of functional diversity which might obscure the identification of important traits that are involved in the assembly process. This allowed us to gain a more detailed picture of the complex processes involved in the assembly of communities. We found clear evidence for dominance of trait underdispersion and could link this to environmental filtering related to moisture conditions on the islands. However, we did not find conclusive evidence for dispersal filtering and we found little evidence for niche partitioning. Although empirical studies such as ours are limited in their ability to link observed pattern to mechanisms, our study makes an important contribution to the identification of key traits that are involved in the assembly processes. Recent advances in coexistence theory suggest that demographic models can be used to quantify the net effect of relative fitness differences (which drive competitive exclusion) and stabilizing niche differences (promoting stable co-existence) (e.g., Levine and HilleRisLambers 2009). However, a caveat is that the knowledge of which traits are involved in coexistence is currently lacking. A promising step forward is therefore to combine trait based approaches with experimental manipulations and demographic models to be able to disentangle different mechanisms involved in community assembly (HilleRisLambers et al. 2012). In this context, by identifying traits that are involved in the assembly process of snail communities, our study may provide a first step to build on in future studies.

## Supporting Information

**Appendix S1.** **Table A1.** Species list. **Table A2.** Average species abundances per island. **Table A3.** Average trait values for each species. **Table A4.** Food types used to calculate the Rao index for diet. **Table A5.** Ecosystem preferences. **Table A6.** Micro-habitat preferences. **Table A7.** Original categories of the fuzzy coded traits. **Table A8.** Recalculations of some of the fuzzy-coded traits. **Table A9.** Environmental variables for each island. **Table A10.** Minimum, maximum, mean and standard deviation of the environmental variables. **Data S1.** Calculation of Rao functional diversity. **Data S2.** Correction of the diversity measures. **Figure A1.** Changes in standard effect sizes across environmental gradients. **Figure A2.** Correlation matrix of the traits.

## References

[b1] Abrams PA, Chen Xin (2002). The evolution of traits affecting resource acquisition and predator vulnerability: character displacement under real and apparent competition. Am. Nat.

[b2] Badie A, Rondelaud D (1985). Contribution a l'etude experimentale de la predation de Cionella lubrica Muller par Nesovitrea hammonis Strom. Ann. Rech. Vet.

[b3] Barker GM, Efford MG, Barker GM (2004). Predatory gastropods as natural enemies of terrestrial gastropods and other invertebrates. Natural enemies of terrestrial molluscs.

[b4] Barker GM, Mayhill PC (1999). Patterns of diversity and habitat relationships in terrestrial mollusc communities of the Pukeamaru Ecological District, northeastern New Zealand. J. Biogeogr.

[b5] Baur A (1991).

[b6] Baur A, Baur B (1993). Daily movement patterns and dispersal in the land snail Arianta arbustorum. Malacologia.

[b7] Baur B, Bengtsson J (1987). Colonizing ability in land snails on Baltic uplift archipelagos. J. Biogeogr.

[b8] de Bello F, Thuiller W, Leps J, Choler P, Clement JC, Macek P (2009). Partitioning of functional diversity reveals the scale and extent of trait convergence and divergence. J. Veg. Sci.

[b9] de Bello F, Lavergne S, Meynard CN, Leps J, Thuiller W (2010). The partitioning of diversity: showing Theseus a way out of the labyrinth. J. Veg. Sci.

[b10] Bowers MA, Brown JH (1982). Body size and coexistence in desert rodents - Chance or community structure. Ecology.

[b11] Brouwers NC, Newton AC (2009). Movement rates of woodland invertebrates: a systematic review of empirical evidence. Insect Conserv. Divers.

[b12] Cain AJ (1977). Variation in the spire index of some coiled gastropod shells, and its evolutionary significance. Philos. Trans. R. Soc. Lond. B Biol. Sci.

[b13] Cain AJ, Cowie RH (1978). Activity of different species of land-snail on surfaces of different inclinations. J. Conchol.

[b14] Cameron RAD, Cook LM (1989). Shell size and shape in Madeiran land snails: do niches remain unfilled?. Biol. J. Linn. Soc.

[b15] Chase JM, Abrams PA, Grover JP, Diehl S, Chesson P, Holt RD (2002). The interaction between predation and competition: a review and synthesis. Ecol. Lett.

[b16] Chiba S (1996). Ecological and morphological diversification within single species and character displacement in Mandarina, endemic land snails of the Bonin Islands. J. Evol. Biol.

[b17] Connor EF, Simberloff D (1979). The assembly of species communities: chance or competition?. Ecology.

[b18] Cook LM (2008). Species richness in Madeiran land snails, and its causes. J. Biogeogr.

[b19] Cornelissen JHC, Lavorel S, Garnier E, Diaz S, Buchmann N, Gurvich DE (2003). A handbook of protocols for standardised and easy measurement of plant functional traits worldwide. Aust. J. Bot.

[b20] Cornwell WK, Ackerly DD (2009). Community assembly and shifts in plant trait distributions across an environmental gradient in coastal California. Ecol. Monogr.

[b21] Day JCL, Dowdeswell WH (1968). Natural selection in Cepaea on portland bill. Heredity.

[b22] Diamond JM (1975). Assembly of species communities.

[b23] Dray S, Dufour AB (2007). The ade4 package: implementing the duality diagram for ecologists. J. Stat. Softw.

[b24] Falkner G, Obrdlik P, Castella E, Speight MCD (2001). Shelled gastropoda of western Europe.

[b25] Freschet GT, Dias ATC, Ackerly DD, Aerts R, van Bodegom PM, Cornwell WK (2011). Global to community scale differences in the prevalence of underdispersed over overdispersed leaf trait distributions in plant assemblages. Glob. Ecol. Biogeogr.

[b26] Fukami T, Bezemer TM, Mortimer SR, van der Putten WH (2005). Species overdispersion and trait underdispersion in experimental plant community assembly. Ecol. Lett.

[b27] Garnier E, Lavorel S, Ansquer P, Castro H, Cruz P, Dolezal J (2007). Assessing the effects of land-use change on plant traits, communities and ecosystem functioning in grasslands: a standardized methodology and lessons from an application to 11 European sites. Ann. Bot.

[b28] Gittenberger E, Groenenberg DSJ, Kokshoorn B, Preece RC (2006). Molecular trails from hitch-hiking snails. Nature.

[b29] Gotelli NJ, McCabe DJ (2002). Species co-occurrence: a meta-analysis of J. M. Diamond's assembly rules model. Ecology.

[b30] Götzenberger L, de Bello F, Bråthen KA, Davison J, Dubuis A, Guisan A (2012). Ecological assembly rules in plant communities—approaches, patterns and prospects. Biol. Rev.

[b31] Hatziioannou M, Eleutheriadis N, Lazaridoudimitriadou M (1994). Food preferences and dietary overlap by terrrestrial snails in Logos area (Edessam Macedonia, Northern Greece). J. Molluscan. Stud.

[b32] Hausdorf B (2000). Biogeography of the Limacoidea sensu lato (Gastropoda: Stylommatophora): vicariance events and long-distance dispersal. J. Biogeogr.

[b33] Hax CL, Golladay SW (1993). Macroinvertebrate colonization and biofilm development on leaves and wood in a boreal river. Freshw. Biol.

[b34] Heller J (1987). Shell shape and land snail habitat in a mediteranean desert. Biol. J. Linn. Soc.

[b35] HilleRisLambers J, Adler PB, Harpole WS, Levine JM, Mayfield MM (2012). Rethinking community assembly through the lens of coexistence theory. Ann. Rev. Ecol. Evol. Syst.

[b36] Hubbell SP (2001). The unified neutral theory of biodiversity and biogeography.

[b37] Ingram T, Shurin JB (2009). Trait-based assembly and phylogenetic structure in northeast Pacific rockfish assemblages. Ecology.

[b38] Jocque M, Field R, Brendonck L, De Meester L (2010). Climatic control of dispersal–ecological specialization trade-offs: a metacommunity process at the heart of the latitudinal diversity gradient?. Glob. Ecol. Biogeogr.

[b138] Jost L (2007). Partitioning diversity into independent alpha and beta components. Ecology.

[b39] Kembel SW, Cowan PD, Helmus MR, Cornwell WK, Morlon H, Ackerly DD (2010). Picante: r tools for integrating phylogenies and ecology. Bioinformatics.

[b40] Kers LE (1978).

[b41] Kleyer M, Dray S, de Bello F, Leps J, Pakeman RJ, Strauss B (2012). Assessing species and community functional responses to environmental gradients: which multivariate methods?. J. Veg. Sci.

[b42] Kraft NJB, Valencia R, Ackerly DD (2008). Functional traits and niche-based tree community assembly in an amazonian forest. Science.

[b43] Lee SC, Silliman BR (2006). Competitive displacement of a detritivorous salt marsh snail. J. Exp. Mar. Biol. Ecol.

[b44] Levine JM, HilleRisLambers J (2009). The importance of niches for the maintenance of species diversity. Nature.

[b45] MacArthur RH, Levins R (1967). The limiting similarity, underdispersion, and overdispersion of coexisting species. Am. Nat.

[b145] MacArthur RH, Wilson EO (1967). The theory of island biogeography.

[b46] Martin K, Sommer M (2004). Relationships between land snail assemblage patterns and soil properties in temperate-humid forest ecosystems. J. Biogeogr.

[b47] Mason NWH, Mouillot D, Lee WG, Wilson JB (2005). Functional richness, functional evenness and functional divergence: the primary components of functional diversity. Oikos.

[b48] Mason NWH, Lanoiselee C, Mouillot D, Wilson JB, Argillier C (2008). Does niche overlap control relative abundance in French lacustrine fish communities? A new method incorporating functional traits. J. Anim. Ecol.

[b49] Mason NWH, Richardson SJ, Peltzer DA, de Bello F, Wardle DA, Allen RB (2012). Changes in coexistence mechanisms along a long-term soil chronosequence revealed by functional trait diversity. J. Ecol.

[b50] Mayfield MM, Levine JM (2010). Opposing effects of competitive exclusion on the phylogenetic structure of communities. Ecol. Lett.

[b150] Miklos I, Podani J (2004). Randomization of presence-absence matrices: comments and new algorithms. Ecology.

[b51] Mitchell CE, Power AG (2003). Release of invasive plants from fungal and viral pathogens. Nature.

[b52] Mouchet MA, Villeger S, Mason NWH, Mouillot D (2010). Functional diversity measures: an overview of their redundancy and their ability to discriminate community assembly rules. Funct. Ecol.

[b53] Naaf T, Wulf M (2012). Plant community assembly in temperate forests along gradients of soil fertility and disturbance. Acta Oecol-Int. J. Ecol.

[b54] Nilsson SG, Bengtsson J, As S (1988). Habitat diversity or area per se? species richness of woody plants, carabid beetles and land snails on islands. J. Anim. Ecol.

[b55] Rondelaud D (1977). Les aptidudes malacophages de quelques Mollusques Zonitoidae et leur intéret dans le control biologique de Lymnaea (Galba) truncatula. Ann. Parasitol. Hum. Comp.

[b56] Rondelaud D (1978). Les effets à long terme d'un controle biologique par prédation. Étude expérimentale de la dynamique des populations de plusieurs espèces de Mollusques. Annales de Parasitologie.

[b57] Schamp B, Horsak M, Hajek M (2010). Deterministic assembly of land snail communities according to species size and diet. J. Anim. Ecol.

[b58] Schilthuizen M, Lombaerts M (1994). Population structure and levels of gene flow in the mediterranean land snail *Albinaria corrugata* (Pulmonata, clausiliidae). Evolution.

[b59] Silva IA, Batalha MA (2008). Species underdispersion into life-forms in a hyperseasonal cerrado in central Brazil. Braz. J. Biol.

[b60] Solem A (1985). Simultaneous character underdispersion and overdispersion in Western Australien land snails. Biol. J. Linn. Soc.

[b61] Stevens VM, Trochet A, Van Dyck H, Clobert J, Baguette M (2012). How is dispersal integrated in life histories: a quantitative analysis using butterflies. Ecol. Lett.

[b62] Stubbs WJ, Wilson JB (2004). Evidence for limiting similarity in a sand dune community. J. Ecol.

[b63] Sutherland GD, Harestad AS, Price K, Lertzman KP (2000). Scaling of natal dispersal distances in terrestrial birds and mammals. Conserv. Ecol.

[b64] Taylor JW (1914). Monograph of the land and freshwater Mollusca of the British Isles.

[b65] Vagvolgyi J (1975). Body size, Aerial dispersal, and origin of pacific land snail fauna. Syst. Zool.

[b66] Vellend M (2010). Conceptual synthesis in community ecology. Q. Rev. Biol.

[b67] Villeger S, Mason NWH, Mouillot D (2008). New multidimensional functional diversity indices for a multifaceted framework in functional ecology. Ecology.

[b68] Violle C, Navas ML, Vile D, Kazakou E, Fortunel C, Hummel I (2007). Let the concept of trait be functional!. Oikos.

[b69] Weiher E, Keddy PA (1995). Assembly rules, null models, and trait dispersion - New questions from old patterns. Oikos.

[b70] Wilson JB, Stubbs WJ (2012). Evidence for assembly rules: limiting similarity within a saltmarsh. J. Ecol.

[b71] Zaret TM (1980). Predation and freshwater communities.

[b72] Zobel M (1997). The relative of species pools in determining plant species richness: an alternative explanation of species coexistence?. Trends Ecol. Evol.

