# Supplementary material for: Underdispersion and overdispersion of traits in terrestrial snail communities on islands
Source: Ecol Evol. 2014 Apr 26;4(11):2090–102. doi: 10.1002/ece3.1084 (PMC4201424; doi:10.1002/ece3.1084)
Supplement: Supplementary file 1 [file ece30004-2090-sd1.docx]

Appendix 1

Table A1 Species list

| **family** | **species** |
| --- | --- |
| Bradybaenidae | *Fruticicola fruticum* (O.F.Müller, 1774) |
| Carychiidae | *Carychium minimum* (O. F. Müller, 1774) |
|  | *Carychium tridentatum* (Risso, 1826) |
| Clausiliidae | *Cochlodina laminate* (Montagu, 1803) |
|  | *Clausilia bidentata* (Ström 1765) |
| Cochlicopidae | *Cochlicopa lubrica* (O. F. Müller, 1774) |
|  | *Cochlicopa lubricella* (Rossmässler, 1834) |
| Discidae | *Discus ruderatus* (W. Hartmann, 1821) |
| Euconulidae | *Euconulus fulvus* (O. F. Müller, 1774) |
|  | *Euconulus alderi* (J.E.Gray, 1840) |
| Gastrodontidae | *Zonitoides nitidus* (O. F. Müller, 1774) |
| Helicidae | *Arianta arbustorum* (Linnaeus, 1758) |
|  | *Helicigona lapicida* (Linnaeus, 1758) |
|  | *Helix pomatia* (Linnaeus, 1758) |
| Hygromiidae | *Euomphalia strigella* (Draparnaud, 1801) |
| Oychilidae | *Nesovitrea hammonis* (Ström, 1765) |
|  | *Nesovitrea petronella* (L. Pfeiffer, 1853) |
| Punctidae | *Punctum pygmaeum* (Draparnaud, 1801) |
| Succineidae | *Succinea oblonga* (Draparnaud, 1801) |
| Valloniidae | *Vallonia costata* (O. F. Müller, 1774) |
|  | *Vallonia pulchella* (O. F. Müller, 1774) |
|  | *Vallonia excentrica* (Sterki, 1893) |
|  | *Acanthinula aculeate* (O. F. Müller, 1774) |
| Vertigidae | *Columella edentula* (Draparnaud, 1805) |
|  | *Columella aspera* (Waldén, 1966) |
|  | *Truncatellina cylindrical* (Férrusac, 1807) |
|  | *Vertigo pusilla* (O. F. Müller, 1774) |
|  | *Vertigo substriata* (Jeffreys, 1833) |
|  | *Vertigo pygmaea* (Draparnaud, 1801) |
|  | *Vertigo ronnebyensis* (Westerlund, 1871) |
|  | *Vertigo angustior* (Jeffreys, 1830) |
| Vitrinidae | *Vitrina pellucida* (O. F. Müller, 1774) |
|  | *Vitrea contracta* (Westerlund, 1871) |

Table A2 average species abundances per island.

|  | Grävlingen | Benklädet | Hargen | Suggan | Rundskär | Hummelskär | St. Askholmen (Prästfjorden) | Högholmen |
| --- | --- | --- | --- | --- | --- | --- | --- | --- |
| *Succinea oblonga* | 17.50 | 0.86 | 1.00 | 0 | 0.17 | 0 | 3.95 | 14.92 |
| *Carychium minimum* | 0 | 0 | 0.14 | 0 | 0 | 0.10 | 0 | 0 |
| *Carychium tridentatum* | 0 | 0 | 0 | 0 | 0 | 0 | 0 | 0 |
| *Cochlicopa lubrica* | 0 | 0 | 0.14 | 1.00 | 4.50 | 0.20 | 4.11 | 0.75 |
| *Cochlicopa lubricella* | 30.67 | 30.57 | 52.00 | 3.86 | 2.21 | 0.30 | 7.47 | 0 |
| *Columella edentula* | 0 | 0 | 0 | 0 | 0 | 0 | 0 | 0.92 |
| *Columella aspera* | 0 | 0 | 0 | 0 | 0 | 0 | 2.14 | 0 |
| *Truncatellina cylindrica* | 3.33 | 0 | 0 | 0 | 0 | 0 | 0 | 0 |
| *Vertigo pusilla* | 4.83 | 3.57 | 39.43 | 2.43 | 2.42 | 0.39 | 12.71 | 14.50 |
| *Vertigo substriata* | 0 | 0 | 7.29 | 0.14 | 4.50 | 1.89 | 3.14 | 0.70 |
| *Vertigo pygmaea* | 0 | 0 | 0 | 0 | 0 | 0 | 0 | 0 |
| *Vertigo ronnebyensis* | 0 | 0 | 0 | 0 | 0 | 0 | 0.45 | 0 |
| *Vertigo angustior* | 0 | 0.14 | 0.67 | 0 | 0 | 0 | 0 | 2.60 |
| *Vitrina pellucida* | 0.33 | 0.86 | 6.57 | 0.14 | 0.25 | 0 | 4.63 | 6.52 |
| *Vallonia costata* | 87.17 | 0.14 | 69.43 | 11.14 | 0.17 | 0 | 0.48 | 14.58 |
| *Vallonia pulchella* | 0 | 0 | 0 | 0 | 0 | 0 | 0.04 | 3.33 |
| *Vallonia excentrica* | 0 | 0 | 0 | 0 | 0 | 0 | 0 | 0 |
| *Acanthinula aculeata* | 0 | 0 | 0 | 8.14 | 0 | 0 | 0 | 0 |
| *Punctum pygmaeum* | 27.50 | 217.14 | 295.57 | 88.29 | 5.08 | 19.07 | 115.40 | 219.42 |
| *Discus ruderatus* | 0 | 0 | 1.57 | 0.43 | 0 | 0.27 | 0.34 | 0 |
| *Vitrea contracta* | 0 | 0 | 0 | 0 | 0 | 0 | 0 | 0 |
| *Nesovitrea hammonis* | 22.00 | 6.43 | 70.43 | 4.57 | 14.00 | 4.67 | 24.64 | 19.58 |
| *Nesovitrea petronella* | 0 | 0 | 7.00 | 0 | 0 | 0 | 1.43 | 0 |
| *Zonitoides nitidus* | 0 | 0 | 0 | 0 | 0.50 | 0 | 0.21 | 0 |
| *Euconulus fulvus* | 0 | 0 | 0 | 1.57 | 1.42 | 1.70 | 1.28 | 0.60 |
| *Euconulus alderi* | 0 | 0 | 0 | 0 | 0 | 0 | 0 | 0 |
| *Cochlodina laminata* | 0 | 1.29 | 2.71 | 0.43 | 0 | 0.50 | 1.30 | 2.25 |
| *Clausilia bidentata* | 0.33 | 2.14 | 2.14 | 0.43 | 0.33 | 1.99 | 0.56 | 1.10 |
| *Fruticicola fruticum* | 0.50 | 0 | 0 | 0 | 0 | 0 | 0 | 1.17 |
| *Euomphalia strigella* | 0.67 | 0.43 | 0.57 | 0 | 0 | 0 | 0.45 | 2.21 |
| *Arianta arbustorum* | 0 | 0 | 0.43 | 0 | 0 | 0 | 0 | 0 |
| *Helicigona lapicida* | 0 | 0 | 0 | 0 | 0 | 0 | 0.45 | 0 |
| *Helix pomatia* | 0 | 0 | 0 | 0 | 0 | 0 | 0 | 0.33 |

Table A2 (continued)

|  | L. Askholmen | Ekholmen | Alholmen | St. Askholmen | Räfsgarn | Gräggen | St. Jungfrun | Kalvholmen | Götön |
| --- | --- | --- | --- | --- | --- | --- | --- | --- | --- |
| *Succinea oblonga* | 0.17 | 1.31 | 0.10 | 2.13 | 5.76 | 0.04 | 0.33 | 0 | 0.04 |
| *Carychium minimum* | 0 | 0.43 | 0 | 14.81 | 0 | 0.54 | 0 | 0 | 0 |
| *Carychium tridentatum* | 0 | 0.05 | 0 | 0 | 0.16 | 0.21 | 0 | 5.92 | 8.21 |
| *Cochlicopa lubrica* | 0 | 3.24 | 1.71 | 7.54 | 0 | 3.10 | 2.29 | 2.31 | 3.43 |
| *Cochlicopa lubricella* | 0.50 | 6.58 | 10.90 | 1.58 | 22.68 | 1.15 | 0.08 | 2.62 | 5.89 |
| *Columella edentula* | 0 | 0.05 | 0 | 0.20 | 0.20 | 0.21 | 0.25 | 0.80 | 0.57 |
| *Columella aspera* | 0 | 0.13 | 0.17 | 0.17 | 0.45 | 0.08 | 1.75 | 0 | 1.06 |
| *Truncatellina cylindrica* | 0 | 0 | 0 | 0 | 0 | 0 | 0 | 0 | 0 |
| *Vertigo pusilla* | 0 | 0.84 | 0.29 | 1.42 | 19.21 | 0.13 | 0.54 | 0 | 0.68 |
| *Vertigo substriata* | 1.00 | 1.36 | 0.13 | 0.74 | 1.09 | 0.54 | 0.63 | 0.22 | 0.59 |
| *Vertigo pygmaea* | 0 | 0 | 0 | 0 | 2.50 | 0 | 0 | 0 | 0 |
| *Vertigo ronnebyensis* | 0 | 0 | 0 | 0 | 0 | 0.50 | 0.63 | 0 | 0 |
| *Vertigo angustior* | 0 | 0 | 0 | 0 | 5.81 | 0 | 0 | 0 | 0 |
| *Vitrina pellucida* | 0.17 | 0.16 | 0.10 | 0.78 | 8.71 | 0 | 1.17 | 0.07 | 0.14 |
| *Vallonia costata* | 0 | 0.05 | 0 | 0.13 | 55.19 | 0.07 | 1.17 | 0.22 | 0.18 |
| *Vallonia pulchella* | 0 | 0.05 | 0 | 0 | 0 | 0 | 0.25 | 0.14 | 1.75 |
| *Vallonia excentrica* | 0 | 0.05 | 0 | 0 | 5.38 | 0 | 0 | 0 | 0.04 |
| *Acanthinula aculeata* | 0 | 0.52 | 0 | 0 | 0 | 0 | 0 | 0 | 3.85 |
| *Punctum pygmaeum* | 20.83 | 14.05 | 33.71 | 35.59 | 86.57 | 9.09 | 2.83 | 9.45 | 9.53 |
| *Discus ruderatus* | 2.67 | 1.53 | 2.57 | 0 | 0.04 | 0 | 0 | 0 | 0.15 |
| *Vitrea contracta* | 0 | 0 | 0 | 0 | 0 | 0 | 0 | 0 | 1.59 |
| *Nesovitrea hammonis* | 24.17 | 18.05 | 14.62 | 23.32 | 8.58 | 11.63 | 9.46 | 11.48 | 11.40 |
| *Nesovitrea petronella* | 0 | 0.87 | 0 | 0 | 0 | 0 | 0 | 0.73 | 1.14 |
| *Zonitoides nitidus* | 0 | 1.57 | 0 | 1.43 | 0 | 2.36 | 0 | 0 | 0 |
| *Euconulus fulvus* | 1.50 | 3.13 | 0.68 | 9.29 | 8.89 | 0.76 | 1.25 | 0 | 1.50 |
| *Euconulus alderi* | 0 | 1.12 | 0 | 1.86 | 1.29 | 0 | 0 | 0 | 0 |
| *Cochlodina laminata* | 0 | 2.48 | 0.29 | 0.13 | 1.57 | 0 | 0 | 0.45 | 0.30 |
| *Clausilia bidentata* | 0 | 0.49 | 0 | 1.16 | 0.36 | 0.08 | 0 | 0.06 | 0.17 |
| *Fruticicola fruticum* | 0 | 0.14 | 0 | 0 | 0.55 | 0.07 | 0.08 | 0 | 0 |
| *Euomphalia strigella* | 0 | 0.29 | 0 | 0 | 2.08 | 0 | 0 | 0.05 | 0 |
| *Arianta arbustorum* | 0 | 0 | 0 | 0 | 0 | 0 | 0 | 0 | 0 |
| *Helicigona lapicida* | 0 | 0.10 | 0.05 | 0 | 0.18 | 0 | 0 | 0 | 0.05 |
| *Helix pomatia* | 0 | 0 | 0 | 0 | 0 | 0 | 0 | 0 | 0 |

Table A3 Average trait values for each species calculated as explained in Table A8.1 and A8.2

|  | number of reproduction periods | max. shell size | shell shape | reproduction mode | number of offspring | age at maturity | inundation tolerance | survival of dry pperiod | humidity prefernece |
| --- | --- | --- | --- | --- | --- | --- | --- | --- | --- |
| *Acanthinula aculeata* | 3 | 1.00 | 2.00 | 1.17 | 0.72 | 2.00 | 1.25 | 2.00 | 2.00 |
| *Arianta arbustorum* | 4 | 4.00 | 2.00 | 0.60 | 1.67 | 3.00 | 3.00 | 2.50 | 2.40 |
| *Carychium minimum* | 6 | 1.00 | 3.00 | 0.67 | 0.82 | 1.00 | 3.00 | 1.00 | 3.00 |
| *Carychium tridentatum* | 5 | 1.00 | 3.00 | 0.67 | 0.82 | 2.00 | 2.33 | 1.50 | 2.40 |
| *Clausilia bidentata* | 2 | 3.00 | 3.00 | 0.50 | 0.86 | 3.00 | 1.33 | 2.00 | 2.00 |
| *Cochlicopa lubrica* | 5 | 3.00 | 3.00 | 1.00 | 0.75 | 1.33 | 3.00 | 1.33 | 3.00 |
| *Cochlicopa lubricella* | 3 | 2.25 | 3.00 | 1.00 | 0.75 | 1.50 | 1.00 | 2.00 | 1.60 |
| *Cochlodina laminata* | 2 | 3.50 | 3.00 | 0.50 | 1.11 | 3.00 | 2.25 | 1.67 | 2.00 |
| *Columella aspera* | 2 | 1.50 | 3.00 | 1.00 | 0.75 | 1.00 | 1.00 | 1.50 | 2.00 |
| *Columella edentula* | 1 | 2.00 | 3.00 | 1.00 | 0.75 | 1.00 | 1.25 | 1.33 | 2.00 |
| *Discus ruderatus* | 3 | 3.00 | 1.00 | 1.20 | 0.71 | 1.75 | 1.25 | 2.33 | 2.25 |
| *Euconulus alderi* | 2 | 2.00 | 2.00 | 1.00 | 0.75 | 1.25 | 3.00 | 1.25 | 3.00 |
| *Euconulus fulvus* | 5 | 1.50 | 2.00 | 1.00 | 0.75 | 1.40 | 1.25 | 1.40 | 2.00 |
| *Euomphalia strigella* | 1 | 3.50 | 2.00 | 0.60 | 1.67 | 2.25 | 1.00 | 2.67 | 1.60 |
| *Fruticicola fruticum* | 2 | 3.75 | 2.00 | 0.60 | 1.67 | 3.00 | 2.67 | 2.25 | 2.00 |
| *Helicigona lapicida* | 3 | 3.75 | 1.00 | 0.75 | 1.60 | 3.00 | 1.00 | 3.00 | 1.50 |
| *Helix pomatia* | 1 | 4.00 | 2.00 | 0.60 | 1.67 | 3.00 | 2.50 | 2.50 | 2.00 |
| *Nesovitrea hammonis* | 3 | 2.00 | 1.00 | 0.75 | 0.80 | 1.50 | 1.50 | 1.50 | 2.50 |
| *Nesovitrea petronella* | 2 | 2.00 | 1.00 | 0.75 | 0.80 | 1.50 | 2.00 | 1.50 | 3.00 |
| *Punctum pygmaeum* | 5 | 1.00 | 1.50 | 1.09 | 0.73 | 1.75 | 2.50 | 2.00 | 2.00 |
| *Succinea oblonga* | 4 | 3.00 | 3.00 | 0.50 | 1.11 | 1.50 | 2.50 | 2.00 | 1.75 |
| *Truncatellina cylindrica* | 2 | 1.00 | 3.00 | 1.00 | 0.75 | 1.00 | 1.25 | 2.00 | 1.25 |
| *Vallonia costata* | 1 | 1.50 | 1.00 | 1.40 | 0.68 | 1.00 | 1.00 | 2.40 | 1.40 |
| *Vallonia excentrica* | 3 | 1.25 | 1.00 | 1.40 | 0.68 | 1.00 | 1.50 | 2.40 | 1.25 |
| *Vallonia pulchella* | 3 | 1.50 | 1.00 | 1.50 | 1.08 | 1.00 | 2.33 | 1.80 | 2.60 |
| *Vertigo angustior* | 2 | 1.00 | 3.00 | 1.00 | 0.75 | 1.00 | 3.00 | 1.00 | 2.40 |
| *Vertigo pusilla* | 2 | 1.00 | 3.00 | 1.00 | 0.75 | 1.00 | 1.50 | 1.50 | 2.00 |
| *Vertigo pygmaea* | 2 | 1.00 | 3.00 | 1.00 | 0.75 | 1.00 | 2.33 | 1.80 | 2.00 |
| *Vertigo ronnebyensis* | 2 | 1.00 | 3.00 | 1.00 | 0.75 | 1.00 | 1.00 | 1.50 | 2.00 |
| *Vertigo substriata* | 2 | 1.00 | 3.00 | 1.00 | 0.75 | 1.00 | 1.33 | 1.50 | 2.60 |
| *Vitrea contracta* | 3 | 1.50 | 1.00 | 1.00 | 0.75 | 1.00 | 1.33 | 1.00 | 2.40 |
| *Vitrina pellucida* | 3 | 2.25 | 2.00 | 0.60 | 1.09 | 1.67 | 2.33 | 1.25 | 2.40 |
| *Zonitoides nitidus* | 4 | 3.00 | 1.00 | 1.33 | 0.69 | 1.50 | 3.00 | 1.00 | 3.00 |

Table A4 Food types used to calculate the Rao index for diet

|  | deciduous. litter | fungi | lichens | mosses | algae | higher plants alive | saprophagous | carnivorous |
| --- | --- | --- | --- | --- | --- | --- | --- | --- |
| *Acanthinula aculeata* | 1 | 0 | 0 | 0 | 1 | 0 | 0 | 0 |
| *Arianta arbustorum* | 0 | 1 | 1 | 0 | 1 | 1 | 1 | 0 |
| *Carychium minimum* | 0 | 0 | 0 | 1 | 1 | 0 | 0 | 0 |
| *Carychium tridentatum* | 1 | 0 | 0 | 1 | 1 | 0 | 0 | 0 |
| *Clausilia bidentata* | 1 | 0 | 1 | 0 | 1 | 0 | 0 | 0 |
| *Cochlicopa lubrica* | 0 | 0 | 0 | 0 | 1 | 0 | 0 | 0 |
| *Cochlicopa lubricella* | 0 | 0 | 0 | 1 | 1 | 0 | 0 | 0 |
| *Cochlodina laminata* | 0 | 0 | 1 | 0 | 1 | 0 | 0 | 0 |
| *Columella aspera* | 0 | 0 | 0 | 0 | 1 | 0 | 0 | 0 |
| *Columella edentula* | 1 | 0 | 0 | 0 | 1 | 0 | 0 | 0 |
| *Discus ruderatus* | 1 | 1 | 1 | 0 | 1 | 0 | 0 | 0 |
| *Euconulus alderi* | 1 | 1 | 0 | 1 | 1 | 0 | 1 | 0 |
| *Euconulus fulvus* | 1 | 1 | 0 | 1 | 1 | 0 | 1 | 1 |
| *Euomphalia strigella* | 0 | 0 | 0 | 0 | 1 | 1 | 0 | 0 |
| *Fruticicola fruticum* | 0 | 0 | 0 | 0 | 1 | 1 | 1 | 0 |
| *Helicigona lapicida* | 0 | 0 | 1 | 1 | 1 | 0 | 0 | 1 |
| *Helix pomatia* | 0 | 0 | 0 | 0 | 1 | 1 | 1 | 0 |
| *Nesovitrea hammonis* | 0 | 0 | 0 | 0 | 1 | 0 | 1 | 1 |
| *Nesovitrea petronella* | 0 | 0 | 0 | 0 | 1 | 0 | 1 | 0 |
| *Punctum pygmaeum* | 1 | 0 | 0 | 0 | 1 | 0 | 0 | 0 |
| *Succinea oblonga* | 0 | 0 | 0 | 0 | 1 | 0 | 0 | 0 |
| *Truncatellina cylindrica* | 0 | 0 | 0 | 0 | 1 | 0 | 0 | 0 |
| *Vallonia costata* | 0 | 0 | 0 | 0 | 0 | 1 | 0 | 0 |
| *Vallonia excentrica* | 0 | 0 | 0 | 0 | 0 | 1 | 0 | 0 |
| *Vallonia pulchella* | 0 | 0 | 0 | 0 | 1 | 1 | 0 | 0 |
| *Vertigo angustior* | 0 | 0 | 0 | 0 | 1 | 0 | 0 | 0 |
| *Vertigo pusilla* | 1 | 0 | 0 | 0 | 1 | 0 | 0 | 0 |
| *Vertigo pygmaea* | 1 | 0 | 0 | 0 | 1 | 0 | 0 | 0 |
| *Vertigo ronnebyensis* | 0 | 0 | 0 | 0 | 1 | 0 | 0 | 0 |
| *Vertigo substriata* | 1 | 0 | 0 | 0 | 1 | 0 | 0 | 0 |
| *Vitrea contracta* | 0 | 0 | 0 | 0 | 1 | 0 | 1 | 0 |
| *Vitrina pellucida* | 1 | 0 | 1 | 0 | 1 | 0 | 1 | 1 |
| *Zonitoides nitidus* | 0 | 0 | 0 | 0 | 1 | 0 | 1 | 1 |

Table A 5 Ecosystem preferences

|  | deciduous forest | scrub | mixed forest | coniferous forest | tall herb | therm. forest frings | unimproved grassland | heathland | coastal dunes | inland dunes | cliff rock | screes/ walls | hedges | fen | reed | water edge |
| --- | --- | --- | --- | --- | --- | --- | --- | --- | --- | --- | --- | --- | --- | --- | --- | --- |
| *Acanthinula aculeata* | 1 | 1 | 0 | 0 | 0 | 0 | 0 | 0 | 0 | 0 | 1 | 1 | 1 | 0 | 0 | 0 |
| *Arianta arbustorum* | 1 | 1 | 1 | 0 | 1 | 0 | 1 | 0 | 0 | 0 | 1 | 1 | 1 | 1 | 0 | 0 |
| *Carychium minimum* | 1 | 0 | 0 | 0 | 1 | 0 | 1 | 0 | 0 | 0 | 0 | 1 | 0 | 1 | 1 | 1 |
| *Carychium tridentatum* | 1 | 1 | 1 | 0 | 1 | 0 | 1 | 0 | 0 | 0 | 1 | 1 | 1 | 1 | 1 | 0 |
| *Clausilia bidentata* | 1 | 1 | 1 | 0 | 1 | 0 | 1 | 0 | 1 | 0 | 1 | 1 | 1 | 0 | 0 | 0 |
| *Cochlicopa lubrica* | 1 | 1 | 1 | 0 | 1 | 0 | 1 | 0 | 1 | 0 | 1 | 1 | 0 | 0 | 1 | 1 |
| *Cochlicopa lubricella* | 1 | 0 | 0 | 0 | 0 | 1 | 1 | 1 | 1 | 1 | 1 | 1 | 1 | 1 | 0 | 0 |
| *Cochlodina laminata* | 1 | 0 | 1 | 1 | 1 | 0 | 0 | 0 | 0 | 0 | 1 | 1 | 1 | 0 | 0 | 0 |
| *Columella aspera* | 1 | 0 | 1 | 1 | 0 | 0 | 0 | 0 | 0 | 0 | 1 | 1 | 0 | 0 | 0 | 0 |
| *Columella edentula* | 1 | 1 | 1 | 0 | 1 | 0 | 1 | 1 | 0 | 0 | 1 | 1 | 0 | 1 | 1 | 0 |
| *Discus ruderatus* | 1 | 0 | 1 | 1 | 0 | 0 | 0 | 0 | 0 | 0 | 0 | 0 | 0 | 0 | 0 | 0 |
| *Euconulus alderi* | 1 | 0 | 1 | 0 | 1 | 0 | 1 | 0 | 0 | 0 | 0 | 0 | 0 | 1 | 1 | 1 |
| *Euconulus fulvus* | 1 | 1 | 0 | 1 | 1 | 0 | 1 | 0 | 1 | 0 | 1 | 1 | 1 | 1 | 1 | 0 |
| *Euomphalia strigella* | 1 | 1 | 0 | 1 | 0 | 1 | 1 | 0 | 0 | 0 | 1 | 1 | 0 | 0 | 0 | 0 |
| *Fruticicola fruticum* | 1 | 0 | 1 | 0 | 1 | 0 | 1 | 0 | 0 | 1 | 1 | 1 | 1 | 0 | 1 | 0 |
| *Helicigona lapicida* | 1 | 0 | 1 | 0 | 0 | 0 | 0 | 0 | 0 | 0 | 1 | 1 | 0 | 0 | 0 | 0 |
| *Helix pomatia* | 1 | 1 | 0 | 0 | 1 | 1 | 1 | 0 | 0 | 0 | 1 | 1 | 1 | 0 | 0 | 0 |
| *Nesovitrea hammonis* | 1 | 1 | 1 | 1 | 1 | 0 | 1 | 0 | 1 | 0 | 1 | 1 | 1 | 1 | 0 | 1 |
| *Nesovitrea petronella* | 1 | 0 | 0 | 0 | 0 | 0 | 0 | 0 | 0 | 0 | 0 | 0 | 0 | 1 | 1 | 1 |
| *Punctum pygmaeum* | 1 | 0 | 1 | 0 | 1 | 0 | 1 | 0 | 0 | 0 | 1 | 1 | 1 | 1 | 1 | 0 |
| *Succinella oblonga* | 0 | 1 | 0 | 0 | 0 | 1 | 1 | 1 | 1 | 1 | 1 | 1 | 0 | 1 | 0 | 1 |
| *Truncatellina cylindrica* | 0 | 1 | 0 | 0 | 0 | 0 | 1 | 0 | 0 | 1 | 1 | 1 | 0 | 0 | 0 | 0 |
| *Vallonia costata* | 1 | 1 | 0 | 0 | 0 | 1 | 1 | 0 | 0 | 1 | 1 | 1 | 1 | 1 | 0 | 0 |
| *Vallonia excentrica* | 0 | 0 | 0 | 0 | 0 | 0 | 1 | 0 | 0 | 1 | 1 | 0 | 0 | 0 | 0 | 0 |
| *Vallonia pulchella* | 0 | 1 | 0 | 0 | 1 | 0 | 1 | 0 | 1 | 1 | 1 | 1 | 1 | 1 | 1 | 1 |
| *Vertigo angustior* | 1 | 0 | 0 | 0 | 0 | 0 | 1 | 0 | 1 | 0 | 0 | 0 | 0 | 1 | 1 | 0 |
| *Vertigo pusilla* | 1 | 1 | 1 | 0 | 0 | 1 | 1 | 0 | 0 | 1 | 1 | 1 | 1 | 0 | 0 | 0 |
| *Vertigo pygmaea* | 1 | 1 | 1 | 0 | 1 | 0 | 1 | 0 | 1 | 1 | 1 | 1 | 1 | 1 | 1 | 0 |
| *Vertigo ronnebyensis* | 0 | 0 | 1 | 1 | 0 | 0 | 0 | 0 | 0 | 0 | 0 | 0 | 0 | 0 | 0 | 0 |
| *Vertigo substriata* | 1 | 0 | 1 | 1 | 1 | 0 | 0 | 0 | 0 | 0 | 1 | 1 | 1 | 1 | 1 | 0 |
| *Vitrea contracta* | 1 | 1 | 1 | 0 | 0 | 0 | 0 | 0 | 0 | 0 | 1 | 1 | 1 | 0 | 0 | 0 |
| *Vitrina pellucida* | 1 | 1 | 1 | 0 | 1 | 0 | 1 | 0 | 1 | 1 | 1 | 1 | 1 | 0 | 0 | 0 |
| *Zonitoides nitidus* | 0 | 0 | 0 | 0 | 1 | 0 | 1 | 0 | 0 | 0 | 0 | 0 | 0 | 1 | 1 | 1 |

Table A6 Micro-habitat preferences

|  | trees | shrubs/ bushes/ saplings | Herb | In moss | timber | forest litter | Herb litter | Stones | Strand debris | Sand | Soil | Bare rock | Root zone | Crevices | Caves |
| --- | --- | --- | --- | --- | --- | --- | --- | --- | --- | --- | --- | --- | --- | --- | --- |
| *Acanthinula aculeata* | 1 | 0 | 1 | 1 | 1 | 1 | 1 | 0 | 0 | 0 | 0 | 0 | 0 | 0 | 0 |
| *Arianta arbustorum* | 1 | 1 | 1 | 0 | 1 | 1 | 1 | 1 | 1 | 0 | 0 | 1 | 0 | 0 | 0 |
| *Carychium minimum* | 0 | 0 | 1 | 1 | 1 | 1 | 1 | 0 | 1 | 0 | 1 | 0 | 0 | 0 | 1 |
| *Carychium tridentatum* | 0 | 0 | 1 | 1 | 1 | 1 | 1 | 1 | 1 | 0 | 1 | 0 | 0 | 0 | 0 |
| *Clausilia bidentata* | 1 | 0 | 0 | 1 | 1 | 1 | 1 | 1 | 0 | 0 | 0 | 0 | 0 | 0 | 0 |
| *Cochlicopa lubrica* | 1 | 1 | 1 | 1 | 1 | 1 | 1 | 1 | 1 | 0 | 0 | 0 | 0 | 0 | 0 |
| *Cochlicopa lubricella* | 0 | 0 | 1 | 1 | 0 | 0 | 1 | 1 | 0 | 1 | 0 | 0 | 0 | 0 | 0 |
| *Cochlodina laminata* | 1 | 0 | 0 | 1 | 1 | 1 | 1 | 1 | 0 | 0 | 0 | 1 | 0 | 0 | 1 |
| *Columella aspera* | 1 | 1 | 1 | 0 | 0 | 1 | 1 | 0 | 0 | 0 | 0 | 0 | 0 | 0 | 0 |
| *Columella edentula* | 1 | 1 | 1 | 1 | 1 | 1 | 1 | 0 | 0 | 0 | 0 | 0 | 0 | 0 | 0 |
| *Discus ruderatus* | 1 | 0 | 0 | 0 | 1 | 1 | 0 | 1 | 0 | 0 | 0 | 0 | 0 | 0 | 0 |
| *Euconulus alderi* | 0 | 0 | 1 | 1 | 1 | 1 | 1 | 0 | 1 | 0 | 0 | 0 | 0 | 0 | 0 |
| *Euconulus fulvus* | 0 | 1 | 1 | 1 | 1 | 1 | 1 | 1 | 0 | 0 | 0 | 0 | 0 | 0 | 0 |
| *Euomphalia strigella* | 1 | 1 | 1 | 0 | 1 | 1 | 1 | 1 | 0 | 0 | 0 | 0 | 0 | 0 | 0 |
| *Fruticicola fruticum* | 1 | 1 | 1 | 0 | 1 | 0 | 1 | 0 | 0 | 0 | 0 | 0 | 0 | 0 | 0 |
| *Helicigona lapicida* | 1 | 0 | 0 | 0 | 1 | 1 | 0 | 1 | 0 | 0 | 0 | 1 | 0 | 0 | 1 |
| *Helix pomatia* | 1 | 1 | 1 | 0 | 1 | 1 | 1 | 1 | 0 | 0 | 0 | 1 | 0 | 0 | 0 |
| *Nesovitrea hammonis* | 0 | 0 | 1 | 1 | 1 | 1 | 1 | 0 | 0 | 0 | 0 | 0 | 0 | 0 | 0 |
| *Nesovitrea petronella* | 0 | 0 | 1 | 1 | 1 | 1 | 1 | 0 | 0 | 0 | 0 | 0 | 0 | 0 | 0 |
| *Punctum pygmaeum* | 0 | 0 | 1 | 1 | 1 | 1 | 1 | 1 | 1 | 0 | 0 | 0 | 0 | 0 | 0 |
| *Succinella oblonga* | 0 | 0 | 1 | 0 | 0 | 1 | 1 | 1 | 1 | 1 | 1 | 0 | 0 | 0 | 0 |
| *Truncatellina cylindrica* | 0 | 0 | 1 | 0 | 0 | 0 | 1 | 1 | 0 | 0 | 0 | 0 | 0 | 0 | 0 |
| *Vallonia costata* | 1 | 1 | 1 | 1 | 0 | 1 | 1 | 1 | 0 | 1 | 0 | 0 | 1 | 1 | 0 |
| *Vallonia excentrica* | 0 | 0 | 1 | 1 | 0 | 0 | 1 | 0 | 0 | 0 | 0 | 0 | 0 | 0 | 0 |
| *Vallonia pulchella* | 0 | 0 | 1 | 1 | 0 | 0 | 1 | 1 | 1 | 0 | 0 | 0 | 1 | 1 | 0 |
| *Vertigo angustior* | 0 | 0 | 1 | 1 | 0 | 0 | 1 | 0 | 1 | 0 | 0 | 0 | 0 | 0 | 0 |
| *Vertigo pusilla* | 1 | 0 | 1 | 1 | 1 | 1 | 1 | 1 | 0 | 0 | 0 | 0 | 0 | 0 | 0 |
| *Vertigo pygmaea* | 0 | 0 | 1 | 1 | 1 | 1 | 1 | 1 | 1 | 0 | 0 | 0 | 0 | 0 | 1 |
| *Vertigo ronnebyensis* | 0 | 1 | 1 | 1 | 1 | 1 | 1 | 0 | 0 | 0 | 0 | 0 | 0 | 0 | 0 |
| *Vertigo substriata* | 1 | 1 | 1 | 1 | 1 | 1 | 1 | 0 | 0 | 0 | 0 | 0 | 0 | 0 | 0 |
| *Vitrea contracta* | 0 | 0 | 1 | 0 | 1 | 1 | 1 | 1 | 0 | 0 | 0 | 0 | 1 | 1 | 1 |
| *Vitrina pellucida* | 0 | 0 | 1 | 1 | 1 | 1 | 1 | 1 | 0 | 1 | 0 | 0 | 0 | 1 | 0 |
| *Zonitoides nitidus* | 0 | 0 | 1 | 1 | 0 | 1 | 1 | 0 | 1 | 0 | 1 | 0 | 0 | 0 | 0 |

Table A7 original categories of the fuzzy coded traits

| **traits** | **categories** | | | | | |
| --- | --- | --- | --- | --- | --- | --- |
| main reproduction period: | Jan/Feb | Mar/Apr | May/Jun | Jul/Aug | Sept/Oct | Nov/Dec |
| maximal shell size: | < 2.5 mm | 2.5 - 5.0 mm | 5-15 mm | > 15 mm |  |  |
| number of offspring: | 1 - 10 | 11-100 |  |  |  |  |
| sexual maturity: | <1 year | 1 year | > 1 year |  |  |  |
| inundation tolerance: | low | moderate | high |  |  |  |
| humidity preference: | dry | moist | wet |  |  |  |
| survival of dry period: | days | weeks | months |  |  |  |
| shell shape: | depressed | globose/ conical | oblong |  |  |  |
| reproduction mode: | cross-fertilization | self-fertilization |  |  |  |  |

**Recalculations of some of the fuzzy-coded traits**

Table A8.1 The trait “number of reproduction periods” was calculated as number of reproduction periods per year from the six main reproduction period categories (Jan/Feb. Mar/Apr. May/Jun. Jul/Aug. Sept/Oct. Nov/Dec).

|  | **main reproduction periods** | | | | | |  |
| --- | --- | --- | --- | --- | --- | --- | --- |
|  | Jan/Feb | Mar/April | May/Juni | July/Aug | Sept/Oct | Nov/Dec | **Number of reproduction periods** |
| Acanthinula aculeata |  |  | 2 | 2 | 1 |  | 3 |

Table A8.2 Example for the calculation of a mean trait value from the fuzzy categories. The fuzzy coding here means that one quarter of the population has a preference for dry sites and three quarters have a preference for moist sites. The mean humidity preference was calculated as the sum of the relative association times the category number: ¼ *1+ ¾ *2 = 1.75.

|  | **humidity preference** | | |
| --- | --- | --- | --- |
|  | dry | moist | wet |
| *category* | 1 | 2 | 3 |
| *Succinella oblonga* | 1 | 3 | 0 |

Table A9 Environmental variables for each island

|  | number of plots | Tot. tree cover | Woody plant diversity | Island area [ha] | Distance to mainland [m] | Distance to the closest large island | Basal area of deciduous trees | Productivity of ground vegetation | Wetness index of ground vegetation | no. of habitats | Leaf dry matter content | Esker ridge |
| --- | --- | --- | --- | --- | --- | --- | --- | --- | --- | --- | --- | --- |
| Kalvholmen | 3 | 93.6 | 30 | 18.30 | 550 | 375 | 83.42 | 14.20 | 1.08 | 5.00 | 281.65 | 0 |
| St. Jungfrun | 2 | 71.5 | 28 | 12.10 | 1650 | 1650 | 53.65 | 3.79 | 0.00 | 4.00 | 285.46 | 0 |
| Gräggen | 4 | 89.7 | 23 | 12.10 | 1300 | 1300 | 92.01 | 4.8 | 0.00 | 3.00 | 284.38 | 0 |
| Högholmen | 2 | 93.0 | 24 | 3.10 | 2750 | 400 | 82.41 | 0.00 | 0.00 | 3.00 | 276.37 | 1 |
| Rundskär | 2 | 88.9 | 22 | 2.60 | 4050 | 450 | 70.80 | 14.7 | 0.38 | 6.00 | 259.70 | 0 |
| Suggan | 1 | 97.5 | 26 | 1.80 | 2650 | 300 | 95.49 | 0.58 | 23.40 | 2.00 | 312.10 | 0 |
| Ekholmen | 3 | 69.7 | 26 | 9.10 | 2500 | 300 | 98.51 | 5.83 | 29.70 | 5.00 | 289.16 | 0 |
| Hargen | 1 | 93.0 | 24 | 1.80 | 2950 | 575 | 98.52 | 2.55 | 0.00 | 4.00 | 267.66 | 1 |
| Grävlingen | 1 | 64.4 | 19 | 0.60 | 2200 | 660 | 88.84 | 4.21 | 0.22 | 5.00 | 274.44 | 1 |
| Benklädet | 1 | 96.7 | 19 | 0.70 | 1200 | 450 | 98.87 | 0.00 | 0.00 | 3.00 | 276.61 | 1 |
| Alholmen | 3 | 96.6 | 22 | 9.40 | 550 | 450 | 79.59 | 8.02 | 0.00 | 2.00 | 296.45 | 0 |
| Räfsgarn | 4 | 95.2 | 26 | 11.40 | 450 | 450 | 91.20 | 9.44 | 0.00 | 5.00 | 289.59 | 1 |
| St Askholmen | 3 | 88.6 | 27 | 12.10 | 600 | 225 | 90.40 | 0.00 | 2.02 | 7.00 | 291.78 | 0 |
| Lilla Askholmen | 1 | 91.4 | 24 | 4.30 | 200 | 140 | 65.34 | 0.00 | 0.27 | 7.00 | 302.98 | 0 |
| Hummelskär | 2 | 94.7 | 18 | 2.10 | 750 | 375 | 74.77 | 0.00 | 0.38 | 4.00 | 293.18 | 0 |
| St Askholmen P | 4 | 90.6 | 29 | 4.50 | 2800 | 1200 | 70.02 | 0.00 | 0.47 | 7.00 | 277.05 | 0 |
| Götön | 4 | 88.5 | 32 | 74.30 | 3400 | 1200 | 68.94 | 8.61 | 0.56 | 5.00 | 298.87 | 0 |

Table A10 minimum, maximum, mean and standard deviation of the environmental variables

|  | min. | max. | mean | standard deviation |
| --- | --- | --- | --- | --- |
| Tot. tree cover | 64.4 | 97.5 | 88.4 | 10.0 |
| Woody plant diversity | 19 | 32 | 25 | 4 |
| Island area | 0.6 | 74.3 | 10.6 | 17.2 |
| Distance to mainland | 200 | 4050 | 1797 | 1196 |
| Distance to closest large island | 50 | 1650 | 629 | 461 |
| Basal area of deciduous trees | 53.65 | 98.87 | 82.52 | 13.44 |
| Productivity of ground vegetation | 0 | 14.7 | 4.5 | 5.0 |
| Wetness index of ground vegetation | 0 | 29.7 | 3.7 | 8.8 |
| no. of habitats | 2 | 7 | 4.5 | 1.6 |
| Leaf dry matter content | 259.7 | 312.1 | 285.73 | 13.21 |

**Calculation of Rao functional diversity**

${\alpha FD}_{\mathrm{Rao}}=\sum_{i=1}^{S} \sum_{j=1}^{S} {d1}_{\mathrm{ij}}p_{\mathrm{ic}}p_{\mathrm{jc}}$ ( Eq. A1)

$\left( + \sum_{i=1}^{S} \sum_{j=1}^{S} {d1}_{\mathrm{ij}}p_{\mathrm{ic}}p_{\mathrm{jc}}+ \sum_{i=1}^{S} \sum_{j=1}^{S} \mathrm{dn}_{\mathrm{ij}}p_{\mathrm{ic}}p_{\mathrm{jc}} \right)_{(multiple traits)}$ (Eq. A2)

${\gamma FD}_{\mathrm{Rao}}= \sum_{i=1}^{S} \sum_{j=1}^{S} d_{\mathrm{ij}}P_{\mathrm{ic}}P_{\mathrm{jc}}\mathrm{with}P_{i}= \frac{\sum_{c}^{n} p_{\mathrm{ic}}}{n}$ (Eq. A3)

α FD is defined as the sum of trait dissimilarities (Gower`s distance. d) among all species pairs in a community weighted by the product of species` (i. j) relative abundances (p). γ FD (Eq. A3) is defined as the sum of trait dissimilarities (distances. d) among all species pairs in the whole region (i.e. all islands). weighted by the product of species` relative abundances (P). β FD is finally defined as the difference between γ FD and the mean α FD (de Bello et al. 2009).

**Correction of the diversity measures based on Jost (2007) and modified for functional diversity by de Bello et al. (2010)**

$\alpha\left( \mathrm{eqv} \right)=\frac{1}{1-\alpha(\mathrm{mean})}$ (Eq. A4)

$\gamma\left( \mathrm{eqv} \right)= \frac{1}{1-\gamma}$ (Eq. A5)

$\beta\left( \mathrm{eqv} \right)= \gamma\left( \mathrm{eqv} \right)- \alpha\left( \mathrm{eqv} \right)$ (Eq. A6)

**Figure A1** Results of regression analyses (/Kruskal Wallis tank sum test for the factor variable ESKER) visualizing significant changes in the standard effect sizes across the environmental gradients. **PROGRO**: productivity of ground vegetation; **DII:** distance to the next largest island; **HAB**: number of habitats; **COVER**: tree cover; **location on esker:** 0 no, 1 yes; **sdp**: survival of dry period; **hpr**: humidity preference; **noo**: number of offspring; **mss**: max. shell size; **ssh**: shell shape; **rep**: reproduction mode; **eco**: ecosystem occurrence; **mic**: microhabitat occurence


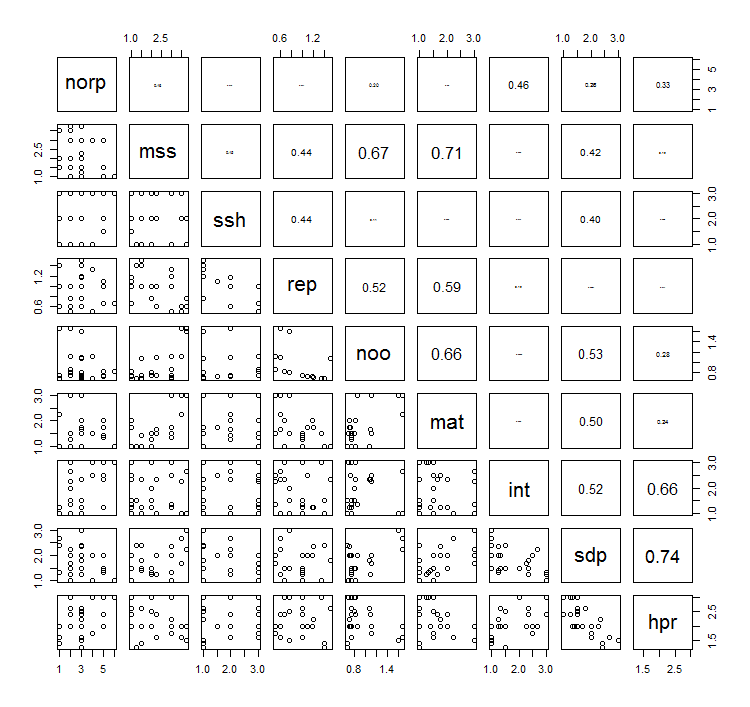


Figure A2 Correlation matrix of the traits. The size of the font represents the strength of the correlation. **norp**: number of reproduction periods. **mss**: max. shell size. **ssh**:shell shape**. rep**: reproduction mode. **noo**: number of offspring. mat: age at maturity. **int**: inundation tolerance. **sdp**: survival of dry period. **hpr**: humidity preference

**Literature**

de Bello, F., W. Thuiller, J. Leps, P. Choler, J. C. Clement, P. Macek, M. T. Sebastia, and S. Lavorel. 2009. Partitioning of functional diversity reveals the scale and extent of trait convergence and divergence. Journal of Vegetation Science **20**:475-486.

de Bello, F., et al. 2010. The partitioning of diversity: showing Theseus a way out of the labyrinth. - J. Veg. Sci. 21:992-1000.

Falkner. G.. Obrdlik. P.. Castella. E.. Speight. M.C.D.(ed.) 2001. Shelled Gastropoda of Western Europe. Verlag der Friedrich-Held-Gesellschaft. Munich.

Jost. L. 2007. Partitioning diversity into independent alpha and beta components. - Ecology 88:2427-2439.
